# Dysbiosis in the Pathogenesis of Pediatric Inflammatory Bowel Diseases

**DOI:** 10.1155/2012/687143

**Published:** 2012-05-20

**Authors:** Donatella Comito, Claudio Romano

**Affiliations:** Pediatric Department, University of Messina, 98125 Messina, Italy

## Abstract

Inflammatory bowel diseases (IBDs) are chronic inflammatory conditions of the gastrointestinal tract that occur in genetically susceptible individuals. Crohn's disease (CD) and ulcerative colitis (UC) are two major types of IBD. In about 20–25% of patients, disease onset is during childhood and pediatric IBD can be considered the best model for studying immunopathogentic mechanisms. The fundamentals of IBD pathogenesis are considered a defective innate immunity and bacterial killing with overaggressive adaptive immune response. A condition of “dysbiosis”, with alterations of the gut microbial composition, is regarded as the basis of IBD pathogenesis. The human gastrointestinal (GI) microbial population is a complex, dynamic ecosystem and consists of up to one thousand different bacterial species. In healthy individuals, intestinal microbiota have a symbiotic relationship with the host organism and carry out important metabolic, “barrier,” and immune functions. Microbial dysbiosis in IBD with lack of beneficial bacteria, together with genetic predisposition, is the most relevant conditions in the pathogenesis of the pediatric IBD.

## 1. Introduction

IBD are chronic inflammations of the small bowel and/or the colon leading to recurrent diarrhea and abdominal pain. Crohn's disease (CD) and ulcerative colitis (UC) are the two main clinicopathological subtypes of IBD. Despite both being chronic and relapsing inflammatory diseases of the bowel, they can be differentiated by the location of the inflammation in the gastrointestinal tract and by the nature of the histological alterations in the intestinal wall. Epidemiology studies suggest that the prevalence of IBD increases in populations and regions with industrialization [1]. Disease onset appears typically in young adulthood (between the age of 25 and 35 years), but in about 20–25% symptoms begin in pediatric population [2]. Complex interactions between immune system, enteric commensal bacteria/pathogens, and host genotype are thought to underlie the development of IBD [3]. An emerging consensus hypothesis is that intestinal dysbiosis (microbial imbalance) may be a trigger for IBD. In children both mucosal immune system and intestinal flora are still in the developmental stage. Taken together it appears that pediatric IBDs represent a specific group of patients with particular gene defects, phenotypic appearance, drug responsiveness, and intestinal immunopathology [4].

In this paper, we will discuss the meaning of dysbiosis in the pathogenesis of pediatric IBD, the weakening of mucosal defences, and the lack of bacterial clearance by macrophages with the result of a loss of tolerance to commensal flora [5, 6].

## 2. Microbial Flora and Intestinal Immune System

The human gut is sterile at birth, but colonization with numerous bacterial species starts immediately after birth, thus generating a resident microbiota characterized by unique bacterial profiles and high interindividual and environmental variation [7]. The adult human microbiota consist of around 10^14^ bacterial cells and up to an estimated 1,000 different bacterial species [8]. Studies have shown that the most abundant bacteria phyla found in healthy human large intestine are Gram-negative Bacteroidetes and Gram-positive low-GC Firmicutes [9]. Microbiota composition varies greatly between individuals, with each individual harbouring a unique collection of bacterial species, which is highly stable over time. The immune regulatory function of the intestinal microbiota consists of priming the mucosal immune system and maintenance of intestinal epithelium homeostasis (Table 1). Studies in germ-free animals have demonstrated that the normal immune function of intestinal mucosa is impaired in the absence of gut microbiota [10]. The “hygiene hypothesis” has been postulated over the years to justify how fundamental lifestyle has changed from one with high to one with low microbial exposure and thus provides an explanation for the higher frequency of IBD [11]. In this condition, the intestinal immune system has smaller Peyer's patches, fewer plasma cells, lower numbers of CD8 intraepithelial lymphocytes with reduced cytotoxicity, and impaired antimicrobial peptide and IgA secretion [12]. The intestinal microbiota are vast and quite diverse at species level. The classification of “normal” microbiota is challenging as each individual possesses a unique collection of microbial species. Firmicutes and Bacteroidetes are the two most predominant bacterial phyla inhabitants in the intestinal tract [13]. The phyla represent the highest taxonomic rank in bacterial classification and are composed of numerous orders, classes, families, and genera with diverse and broad metabolic, ecological, pathogenic, and symbiotic properties [14]. The description of phylum profiles has only limited biological relevance for understanding host-microbe interactions. Evidence suggests that commensal bacteria play a role in maintaining the integrity of the intestinal epithelium [15]. Intestinal epithelial cells (IECs) provide a physical barrier between luminal microbes and underlying intestinal tissues to control defence and tolerance. IECs express pattern recognition receptors (PRRs) and can recognize microbial pathogen-associated molecular patterns (PAMPs) and respond to intestinal microbes through secretion of cytokines and antimicrobial proteins and up-regulation of surface molecules that mediate intercellular interactions [16]. Peterson et al. have shown that the presence of IgA reduces intestinal proinflammatory signals and drives diversity in gut microbiota [17]. A defective antibacterial, genetically driven barrier allows translocation and regulation of the microbiota. Commensal bacteria can have an anti-inflammatory effect on the developing immune system; for example, in the uterus, T-helper type 2 response is predominant [18]. With gut colonization, a balance between T-helper types 1 and 2 is established to prevent the development of allergic food reactions, and to establish a T-helper type 3 response that provides tolerance to oral protein antigens. Feeding allows for antigenic stimulation and bacterial colonization of the gut. This is required for the development of IgM- and IgA-producing plasma cells in the intestinal lamina propria [19]. Breast milk provides passive protection with antibacterial components such as IgA, lysozyme, and lactoferrin, promotes the development of commensal flora rich in bifidobacteria, and decreases colonization with potential pathogens. Alterations in the normal development of the immune system can lead to chronic disease states. Antibiotic use in the neonatal period and infancy can interfere with the development of a healthy commensal flora and may result in subsequent allergic disease or inflammatory conditions of the intestinal tract (irritable bowel syndrome and IBD) [20]. 

In healthy conditions, balanced mechanisms regulate the host's immunological tolerance to the continuous stimulus of resident gut microbiota and their metabolic end products [21]. Hildebrand et al. have shown that pneumonia prior to age 5 years, but not later, and consequent and frequent use of antibiotics were associated with subsequent high risk of CD, and this may represent either susceptibility or causation. The results confirm that early exposures to antibiotics influence immune function through disruption of bowel colonization [22].

## 3. Microbiota in IBD

The theory is discussed that IBDs represent the consequence of the loss of immunological tolerance against autologous flora. Supporting this theory are a limited number of human studies and a large number of studies in animal models. It is assumed that the presence of bacteria is essential for the development of experimental IBD in most models [23]. Commensal flora appears to exacerbate rather than directly cause disease [24]. In IBD patients, not only is the quantity of commensal bacteria reduced but also the quality of microbiota composition is altered, with reduction of Firmicutes and Bacteroidetes. As a consequence of this dysbiosis, the relative abundance of Enterobacteriacae is increased in IBD patients compared to healthy controls, although their absolute numbers remained unaltered [25]. These findings are present also in several studies, which have observed decreased clostridia concentrations, although not always accompanied by a decrease in Bacteroides [26]. Macfarlane et al. revealed aberrancies in *Bifidobacterium* populations in rectal biopsies from IBD patients with significant reductions of the counts [27]. Zhang et al. have shown that bacterial diversity of lactobacilli is present in ulcerated tissue compared to nonulcerated tissue in the same UC individuals [28]. 

On the other hand, the number of mucosal adherent bacteria, such as invasive *E. coli*, or Proteobacteria, such as Enterobacteriaceae are increased (Table 2). The possibility that IBDs are a chronic inflammatory response directed against microbial agents has been considered in UC and CD. Several infectious agents, including *Mycobacterium avium* subspecies *paratuberculosis* (MAP), adherent invasive *E. coli*, *Yersinia*, and *Pseudomonas* have been implicated as triggering agents of CD [29]. Research has excluded many microorganism including salmonella, campylobacter jejuni, *clostridium* difficile, adenoviruses, rotaviruses, and mycoplasma as primary etiological agents, although some may be implicated in relapses of CD [30]. One agent that raised a great deal of controversy is *Mycobacterium avium* subspecies *paratuberculosis* (MAP), which, for many years, was considered a possible etiologic agent [31]. MAP has been the most enduring infectious candidate to be proposed as a causative agent of CD although its role in etiology of disease has often been questioned. Prevalence studies of MAP in CD patients from many countries worldwide have reported widely divergent results ranging from 0% to 100%. The possible role of MAP in CD has also been supported by the identification of MAP DNA using IS900 polymerase chain reaction (PCR) analysis of media inoculated with peripheral blood mononuclear cells (PBMCs) from patients [32]. Kirkwood et al. described a comprehensive investigation into the presence of MAP in intestinal tissue and PBMC from 142 children presenting with initial symptoms of IBD prior to treatment. The final diagnoses included CD (62 children), UC (26 children), and non-IBD (54 children). There was evidence of MAP infection in biopsy tissue and/or PBMC in a total of 45% of children with CD, 35% of children with UC, and 11% of non-IBD children. The presence of viable MAP in 4/10 CD patients was confirmed by isolation of MAP from biopsy specimens. These results support the hypothesis that MAP infection of intestinal tissue, perhaps associated with bloodborne spread, may be implicated especially in the pathogenesis of pediatric CD [33]. No significant correspondence was found between CD-associated NOD2 polymorphisms, especially in ileal CD, and MAP infection [34]. Recently, another microorganism, *Escherichia coli*, has been under investigation and associated with ileal CD [35], but there is no evidence that antibiotic treatment against coliforms is efficacious in curing IBD patients. In a number of different mouse models of colitis, it was possible to prevent colitis by raising the mice under germ-free conditions. The hypothesis was developed that physiologic intestinal flora is no longer tolerated in IBD. Since 2001, genom-wide association studies (GWAs) have revealed more than 30 genes that are associated with IBD [36]. Among the identified targets are genes that play an important role for immunological cell-cell interactions and signalling, such as tumor necrosis factor (TNF), TNF-receptor 1 (TNFR1), the interleukin-23 receptor (IL23R) [37], or interleukin-12p40 (IL12B). More importantly, there are genes involved in the immune response to bacteria, such as nucleotide oligomerization domain 2 (NOD2) and the toll-like receptor 4 (TLR4), as well as the autophagy genes autophagy-related like 1 (ATG16L1) and immunity-related GTPase family M (IRGM) [38]. The variants of ATG16L1 and IRGM autophagy genes cause a defective capacity to process cell degradation products as well as bacteria and to eliminate proinflammatory stimuli [39]. Three mutually exclusive theories have been proposed concerning the implication of bacteria in pathogenesis of IBD, such as an involvement of persistent pathogen, an abnormally permeable mucosal barrier leading to excessive bacterial translocation and a breakdown in the balance between putative “protective” as against “harmful” intestinal bacteria which can promote inflammation. Bacteria colonizing the gut mucosa have the ability to strongly adhere to intestinal epithelial cells (IECs), to invade IECs by a mechanism involving actin polymerisation and microtubule recruitment and to induce granuloma formation *in vitro *[40]. Based on the pathogenic group, this type of *E. coli *was defined and named AIEC for adherent-invasive *E. coli *(AIEC). AIEC strains were found to be highly associated with ileal mucosa in CD patients, suggesting that there are specific alterations to the ileal epithelial cells in patients with CD that allow AIEC adhesion. The receptor involved in AIEC colonization, and abnormally expressed on ileal mucosa in 35% of CD patients, was characterized as being the carcinoembryonic antigen-related cell adhesion molecule (CECAM6). In pediatric population, genetics plays an even greater role in disease onset and susceptibility. It does appear, however, that the NOD2 gene is similarly present in 30%–35% of both adult and pediatric CD patients. Although the true pathogenic role of NOD2 in CD remains unknown, it is an important gene involved in innate immunity which lends support to the notion that genetically determined defects in innate, and likely adaptive immunity, alter the way of interaction of mucosal immune system with resident bacterial flora [41]. This dysregulated interaction leads to the adaptive immune response, responsible for the chronic inflammatory lesions, and is more evident in pediatric-age-onset IBD [42].

## 4. Dysbiosis in IBD: Cause or Effect of the Mucosal Inflammation?

In IBD, dysbiosis could be a key factor in the immunopathogenesis of IBD by disrupting the host immune defences against commensal flora microbes at the mucosal border [43]. Increased paracellular intestinal mucosal barrier has long been recognized in IBD with abnormalities in both its structural integrity and mucus barrier functions [44]. Sewell et al. have hypotized that the penetration of gut luminal contents into the altered bowel wall impaired clearance of this material by the innate immune response and propagation of a secondary inflammatory reaction by the adaptive immune system [45]. Bacterial clearance is also altered in IBD, and an interaction between NOD2 and the autophagy system has been elucidated. Frank et al. performed a genotype-phenotype correlation and gene-environment interaction study of IBD patients. The results show that disease phenotype NOD2 composite genotype (Leu1007fs, R702W, G908R alleles) and ATG16L1 genotype (T300A allele) were significantly associated with shifts in microbial compositions with reduced bacterial diversity [46]. Specifically, members of the Lachnospiraceae family (Firmicutes phylum) and Bacteroidales (bacterial order) were depleted in a subset of IBD samples, with a concomitant increase in 16S rRNA sequences of Proteobacteria and Actinobacteria [47]. As a consequence of this dysbiosis, the relative abundance of Enterobacteriaceae was increased in IBD patients compared to healthy controls, although their absolute numbers remained unaltered. More importantly, this study confirmed that *Faecalibacterium prausnitzii*, a member of the Lachnospiraceae family (clostridial cluster IV and IXa), was reduced in the mucosa of IBD patients [48].

This abnormal microbiota composition shifts complex interactions that occur between microbes and host and its metabolic, trophic, and protective functions, such as immunomodulatory stimulation, strengthening epithelial barrier integrity [49]. In particular, *Clostridium* and Bacteroides species reduction cause reduction of butyrate and short-chain fatty acid production. *F. prausnitzii *have anti-inflammatory and anticolitic properties. Overgrowth of a class of microorganisms referred to as sulphate-reducing bacteria (SRB), observed in UC patients, produces substances which are toxic to colonocytes and blocks protective mechanisms in intestinal mucosa [50]. Numerous studies have analysed microbial compositions in individuals with IBD compared to healthy controls analyzing stool samples, but it is well accepted that microbial populations from stool differ from those associated with the mucosa [51–54]. 

In recent months, however, researchers have been working to characterize the gut microbiota also in pediatric IBD. Richness, evenness, and biodiversity of the gut microbiome were remarkably reduced in 27 children with severe UC compared with healthy controls, and this could include the lack of response to steroid therapy [55].

Darfeuille-Michaud et al. showed that in patients with IBD there was abnormal colonization of the ileal mucosa by AIEC bacteria that induced the release of high amounts of TNF*α* without leading to host cell apoptosis and with potential ability to induce persistent intestinal inflammation [56]. Some years later, the presence of adhesive invasive bacterial strains was confirmed in a pediatric population with IBD, in inflamed intestinal tissue [57].

**Figure 1:**
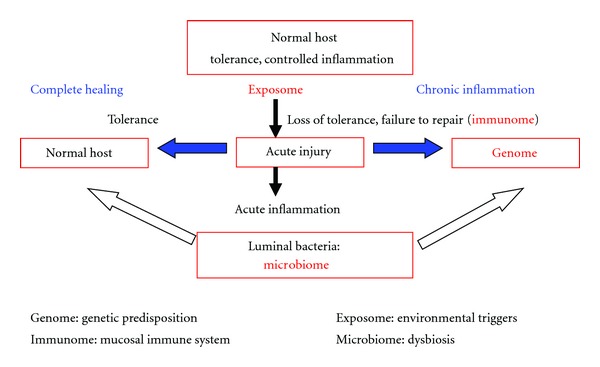
Pathogenesis of IBD: host response and loss of tolerance in intestine.

It is possible to suggest that microbiota and microbiome are different in different sites of inflamed or non-inflamed gut with loss of tolerance and defective in the production or function of antibacterial peptides, such as defensins by the Paneth cells. There is some evidence that alpha-defensin production is reduced in ileal CD [58] and that, in colonic CD, there is reduced mucosal antimicrobial activity with consistently low antibacterial peptide expression [59]. This quantitative and/or qualitative alteration leads to lower levels of defensins in the numbers and type of intestinal microbiota composition and could promote intestinal inflammation. These alterations cause loss of tolerance to commensal flora and to amplification and maintenance of inflammatory response to intestinal pathogens (Figure 1). In trying to establish a pathogenic role of dysbiosis in IBD, microbial imbalance triggers a range of mechanisms with reduced intraluminal levels of butyrate, with downregulation of epithelial tight junction protein expression and increased epithelial permeability [50]. Killing of bacteria reaching the lamina propria, through the “leaky” epithelium, is also impaired by a genetically predisposed defective phagocytosis by macrophages. Ineffective bacterial clearance leads to excessive TLR stimulation, secretion of proinflammatory cytokines, and activation of innate and T-cell mediated immune responses. In summary, defective killing of phagocytosed organisms, decreased secretion of antimicrobial peptides, increased mucosal permeability, or defective excretion of xenobiotic materials could result in an overwhelming stimulation of adaptive immune responses and loss of immunologic tolerance to commensal bacterial antigens [60]. This disrupted mechanism of tolerance in epithelial cells may recognize dysbiosis as a *primum movens*. Despite these observations, it is not clear if gut microbial dysbiosis is a cause or a consequence of inflammatory disease [61] as these studies differ in distinct source of microbes and analytical methods. 

## 5. Conclusions and Perspectives

There have been new findings in identifying the pathogenesis of IBD over the last year, but environment, genetic makeup, commensal flora, and immune response can be considered the key factors. Dysbiosis can be considered an important pathogenetic factor with advancement growth of invasive pathogenic bacteria. It can also facilitate bacterial translocation through the intestinal mucosa barrier to the mesenteric lymph nodes. Analysis of the microbiota of CD and UC has so far resulted in diverging views of the importance of the particular bacteria in pathogenesis of IBD. Not only is the quantity of commensal bacteria in the IBD intestine reduced but also the quality and diversity of the commensal composition are altered. On the other hand, the number of mucosal adherent bacteria, such as invasive *E. coli*, or Proteobacteria, such as Enterobacteriaceae, is increased, resulting in the so-called state of “dysbiosis.” This condition may have a pathogenetic role which is more important in pediatric IBD, where interaction with genetic predisposition is more significant.

Further studies are needed to better define the true composition of the microbiota in patients with IBD and to understand if dysbiosis is a predisposing condition or a consequence of chronic intestinal inflammation. 

## Figures and Tables

**Table 1:** The effect of commensal bacteria on the development of the immune system.

Inhibits epithelial NF-kB activation and inflammatory gene expression	
Activates CD4 cells in Peyer's patches	
Activates CD8 or natural killer cells in intraepithelial leukocyte spaces	
Increases numbers of T and B cells, including CD86-positive cells	
Organizes the special relationships between T, B, and dendritic cells in the Peyer's patches	
Increases the numbers of microfold cells	
Increases IgA producing B cells	
Hypertrophies Peyer's patches and the development of germinal centers	

**Table 2:** Composition of commensal microflora in IBD [48].

Increased	Reduced
*E. coli*	Firmicutes
Proteobacteriace	Bacteroidetes
Enterobacteriacae	*Clostridium* ix and iv groups
Sulphate-reducing bacteria	*Bifidobacteria*
